# Hydrolyzed Collagen as a Potentially Functional Ingredient and Optimizer of Technological Properties of Chocolate‐Flavored Dairy Dessert

**DOI:** 10.1111/1750-3841.70636

**Published:** 2025-10-31

**Authors:** Maria Eduarda Marques Soutelino, Bianca Cristina Rocha de Oliveira, Louise de Aguiar Sobral, Fábio Jorge de Vasconcellos, Eliane Teixeira Mársico, Erick Almeida Esmerino, Adriano Gomes da Cruz, Adriana Cristina de Oliveira Silva

**Affiliations:** ^1^ Department of Food Technology School of Veterinary Medicine Fluminense Federal University (UFF) Niterói Rio de Janeiro Brazil; ^2^ School of Chemistry Federal University of Rio de Janeiro (UFRJ) Rio de Janeiro Rio de Janeiro Brazil; ^3^ Department of Food Federal Institute of Education Science and Technology of Rio de Janeiro (IFRJ) Rio de Janeiro Brazil

**Keywords:** centesimal composition, check‐all‐that‐apply (CATA), dairy product, microstructure, protein enrichment, rheology, scanning electron microscopy, sensory acceptance

## Abstract

It has been reported that hydrolyzed collagen (HC) can provide important functional and technological properties in food products. However, its application in chocolate‐flavored dairy desserts remains underexplored, representing a significant scientific gap. Six chocolate‐flavored functional dairy desserts with varying levels of HC (F1—0%, F2—1%, F3—3%, F4—6%, F5—9%, F6—12%) were evaluated for composition, color, rheology, microstructure, and sensory aspects. Protein content increased with the addition of HC, whereas moisture, fat, and carbohydrates decreased (*p* < 0.05). Luminosity (L*) decreased significantly with the addition of HC, and F6 demonstrated the most pronounced color changes. Microstructural analysis revealed structural differences in the gel formed in F1, F2, and F6, whereas lactose crystal formation was evident at 3% HC. Rheologically, F1 exhibited the most pseudoplastic behavior (0.582) and F6 the least (0.738). The highest consistency index was observed in F5 (59.3 Pa s*^n^*). Sensory analysis favored F2 and F3 for attributes such as creaminess and chocolate flavor, whereas F5 had the lowest acceptance. This study is innovative as it is the first to comprehensively characterize the impact of HC on chocolate‐flavored dairy desserts, providing scientific evidence on its technological and sensory implications.

**Practical Applications:**

This research indicates that hydrolyzed collagen has demonstrated potential as an ingredient for the dairy industry, allowing the development of potentially functional dairy desserts with higher protein content, lower fat and carbohydrate content, as well as optimized texture and consistency. In addition, concentrations of 1%–3% proved to be ideal for maintaining sensory acceptance. This innovation meets the growing demand for healthy foods and can be applied to the creation of differentiated lines of dairy desserts with nutritional, functional, and technological appeal in the market.

## 1 Introduction

The functional food market has been gaining prominence in recent years due to the greater concern for the relationship between diet and food (Nguyen and Phan 2022). In addition to basic nutritional functions, these products can generate other benefits for the body, such as reducing the incidence of chronic diseases and increasing immunity (Rocha et al. 2023; Jacquier et al. 2024). At the same time, there is also a greater appeal for products reduced in fat and sugar related to reducing the incidence and progression of cardiovascular diseases, obesity, and diabetes (Zyriax and Windler 2023). Hydrolyzed collagen (HC) is an excellent functional ingredient, as it can regulate blood pressure (Shori et al. 2021), prevent premature aging (Zhao et al. 2021), in addition to having antioxidant, anti‐inflammatory, antimicrobial (Chotphruethipong et al. 2020), and bone and cartilage regeneration activities (Vasserman et al. 2020).

Due to its high protein content, typically ranging from 84% to 90%, HC can also be used as an essential ingredient in food formulation, as a colloidal stabilizer, emulsifier, foaming agent, and biodegradable film former, positively influencing technological and quality parameters (Gómez‐Guillén et al. 2011; Sousa et al. 2017). The use of HC has been studied in several dairy products such as fermented milk (León‐López et al. 2020; Znamirowska et al. 2020), dairy beverages (Gerhardt et al. 2013), and ice cream (Li et al. 2015), demonstrating significant improvements in aspects such as texture, viscosity, stability, sensory acceptance, and nutritional aspects (Soutelino et al. 2023). However, studies aiming at applying HC in dairy desserts are still scarce in the literature.

Dairy desserts are products that are highly accepted by consumers and come in several flavors and variations (Jahromi and Niakousari 2018; Kuriya et al. 2020), in addition to being sources of essential nutrients such as proteins, calcium, and vitamin D (Yilmaz‐Ersan et al. 2019). Regarding their formulation, these products must have at least 50% of ingredients of dairy origin (Official Gazette of the Federative Republic of Brazil, Ministry of Agriculture, Livestock, and Supply 2020) and others that can play a fundamental role in their texture, flavor, and nutritional quality. Among the different types of dairy desserts, chocolate‐flavored is one of the most consumed and has the greatest sensory acceptance (Morais et al. 2015). The cocoa powder in formulations with this flavor also has high nutritional value (Martín and Ramos 2017) and phenolic compounds that add sensory and functional attributes (Zimmermann and Ellinger 2020).

Considering that HC can modify various technological and structural characteristics of dairy products (Soutelino et al. 2023), it was hypothesized that its application in a dairy dessert could result in a more nutritious product, with better technological attributes and greater sensory acceptance. However, no study has evaluated the effects of HC on reduced‐fat, reduced‐sugar chocolate‐flavored dairy desserts. This matrix remains underexplored in the literature, despite its widespread acceptance and potential for nutritional and technological improvements. Integrated approaches that simultaneously consider physicochemical, microstructural, rheological, and sensory aspects are also scarce. Therefore, this study aimed to analyze the impact of different HC concentrations on these properties, contributing new data for the formulation of healthier, technologically viable, and sensorially appealing products.

## 2 Materials and Methods

### 2.1 Processing of Dairy Dessert

All ingredients were obtained from local stores in Rio de Janeiro, Brazil. The formulation for processing dairy desserts was followed according to the methodology described by Morais et al. (2015) and Furlán and Campderrós (2017). Molico (Rio de Janeiro, Brazil) skimmed UHT milk (54%), Piracanjuba (Goiânia, Brazil) UHT light cream with 17% fat (25%), Molico (Rio de Janeiro, Brazil) skimmed milk powder (15%), Nestlé cocoa powder (Rio de Janeiro, Brazil) (5%), Adicel (Belo Horizonte, Brazil) xanthan gum (0.2%), Dr. Decker (Towson, United States) vanilla essence (0.2%), Stevia Linea (Anápolis, Brazil) sweetener (0.1%), and Adicel (Belo Horizonte, Brazil) potassium sorbate (0.05%) were used. HC Nutrify (São Paulo, Brazil) was added in five different concentrations, 1%, 3%, 6%, 9%, and 12% (w/w), corresponding to F2, F3, F4, F5, and F6 samples, respectively. The sample without adding HC was identified as F1 (control). The treatments were determined according to the recommended daily HC supplementation dose (0.2 g/kg) (Tanaka et al. 2009).

Except for the vanilla essence, the ingredients were homogenized and heated at 85°C for 5 min to pasteurize and hydrate the gum. Afterward, the samples were cooled to 40°C to add the vanilla essence, followed by packaging in 100 mL plastic containers with lids and storage at 5°C for later analysis.

### 2.2 Physicochemical Analysis

#### 2.2.1 Proximate Composition

The HC protein and ash content (%) were previously determined, and the moisture, protein, fat, ash, and carbohydrate content of the dairy dessert samples were performed in triplicate on Day 1, according to the methods described in the “Official Methods of Analysis.” Moisture was determined by oven drying to constant weight, whereas protein content was obtained by the Kjeldahl method, which quantifies total nitrogen. Fat was analyzed by extraction with organic solvents using the Soxhlet method, and ash content was determined by incineration in a muffle furnace at 550°C. Finally, carbohydrate content was estimated by difference, subtracting moisture, protein, fat, and ash values from the total matter (AOAC International 2010).

#### 2.2.2 Color Measurement

Color measurement was performed in triplicate on Day 1 according to the methodology described Rocha et al. (2022), using a portable colorimeter (CR‐410, Minolta Sensing Konica Inc., Tokyo, Japan). The results were obtained according to the CIE Lab* coordinates, corresponding to L* (lightness), a* (green (−)/red (+) chromaticity), and b* (blue (−)/yellow (+). The chroma (C*) value determines the difference between a hue and a gray color and was calculated according to the following equation:

(1) $$C^{*}=\sqrt{a^{*2}+b^{*2}}$$

The hue angle (*h*) is an attribute related to the difference in absorbance at different wavelengths. It was calculated according to the following equation:

(2) $$h^{*}=\mathrm{ta}n^{-1\;}\;\left(\frac{b}{a^{*}}\right)$$

### 2.3 Microstructural Analysis

The microstructural analysis was performed according to the methodology described by Vianna et al. (2019). The 1 cm^3^ samples of dessert were fixed in 2% osmium tetroxide, deposited on coverslips under aluminum stubs with silver epoxy, and coated with gold under vacuum using a coaster (Bal‐Tec SCD 050, Balzers, Liechtenstein). A scanning electron microscope (SEM) (Zeiss evo ma10, Oberkochen, Germany) was used to observe them in five fields.

### 2.4 Rheological Analysis

The rheological assays were performed in a controlled stress rheometer model MCR 501 (Anton Paar Instruments, Canada) using a stainless steel plate‐plate geometry with a diameter of 50 mm. A gap of 0.103 mm was used, and the temperature was maintained at 10°C by a Peltier system.

The flow curve assays were performed by a sweep of the shear rate from 0.1 to 100 s^−1^. The data obtained from the flow curves were fitted to the Herschel–Bulkley model by nonlinear regression analysis using STATISTICA 8.0 (Statsoft, Tulsa, OK, USA):

(3) $$\sigma =\sigma_{0}+k\;{\dot{\gamma }}^{n}$$

where *σ* is the shear stress (Pa), *σ*_0_ is the yield stress (Pa), *k* is the consistency index (Pa s*^n^*), *n* is the flow behavior index (dimensionless), and $\dot{\gamma }$ is the shear rate (s^−1^).

To determine the linear viscoelastic portion, a strain amplitude sweep test (0.01%–100%) was previously performed at a fixed frequency of 6.28 rad/s (data not shown). The dynamic oscillatory assays were carried out within the linear viscoelasticity domain by performing frequency sweeps in the range of 0.1 to 100 rad/s at a constant strain amplitude of 1%. The parameters storage modulus (*G*′), loss modulus (*G*″), and loss tangent (tan *δ* = *G*″/*G*′) were determined for all samples.

### 2.5 Sensory Analysis

The sensory evaluation was carried out with 120 dairy consumers, male and female, aged between 20 and 62 years old and randomly recruited from students and collaborators of the Veterinary School of the Fluminense Federal University (UFF). The acceptance test and the check‐all‐that‐apply (CATA) (Soutelino et al. 2022) were performed using the Compusense Cloud software (CompuSense Inc, Ontario, Canada). The six samples of dairy dessert were coded with three random digits and served in 10 g portions at 5°C in 50 mL plastic cups, in a monadic and randomized manner, in individual booths and a room with fluorescent lighting, following international standards (ISO 1988). The evaluators received mineral water and small neutral snacks to cleanse the palate between samples, with intervals of approximately 1 min to minimize the effects of sensory fatigue. The privacy rights of the participants were preserved, and consent for experimentation was obtained.

#### 2.5.1 Acceptance Test

The overall liking was performed using a structured 9‐point hedonic scale, ranging from “I disliked it very much” to “I liked it very much” (Soutelino et al. 2022).

#### 2.5.2 Check‐All‐That‐Apply (CATA)

The CATA questionnaire (Soutelino et al. 2022) was performed to define the sensory profile of the samples and an ideal product (dairy dessert). The 18 terms used were defined by 25 evaluators (consumers) from the UFF, following Kelly's Repertory Grid Method (Ares et al. 2015). The sensory attributes of appearance (color, shine, presence of particles, apparent consistency), flavor/taste (sweet, dairy, chocolate, bitter, and coffee), aroma (sweet and coffee), and texture (homogeneous, viscous, elastic, creamy, consistent, and with the presence of lumps) were used to describe the sensory profile of the samples. The presentation order followed Willian Latin's experimental square (Ares et al. 2015), balanced between the sample and tasters.

### 2.6 Statistical Analysis

The physicochemical analysis and acceptance data were submitted to ANOVA, considering the dessert formulation (F1–F6) as the independent variable and the physicochemical parameters and acceptance scores as dependent variables; replicates were included to estimate intragroup variability, and multiple comparisons were performed by Tukey's test (*p* < 0.05) using XLSTAT 2015.3 (Addinsoft, Paris, France). For rheology, the data obtained from the flow curves were adjusted to the Herschel–Bulkley model by nonlinear regression analysis using STATISTICA 8.0 (Statsoft, Tulsa, OK, USA). For the CATA analysis, a contingency table was obtained, determined by the frequency of citation of each sensory attribute by counting the number of participants who used the term to describe the sample subjected to correspondence analysis (CA) (Esmerino et al. 2017), penalty analysis, and Cochran's *Q*‐test (Oliveira et al. 2017), associating affective data with descriptive data.

### 2.7 Ethical Guidelines

All procedures performed in this research are in accordance with the laws and institutional guidelines of the Research Ethics Committee of the Federal Institute of Education, Science and Technology, having been approved on June 13, 2022, under registration number 58961422.6.0000.5268. Informed consent was obtained from each subject before they participated in the sensory analysis. All procedures performed were in accordance with the laws and institutional guidelines applicable to research involving human subjects.

## 3 Results and Discussion

### 3.1 Physicochemical Analysis

#### 3.1.1 Proximate Composition

Verifying the protein content of HC is a fundamental aspect as it allows greater reliability, standardization of samples, and guarantee of technological and nutritional benefits. The protein and ash content of HC used in the formulations of dairy desserts were 92.8% and 0.8%, respectively, corroborating what was described by Gómez‐Guillén et al. (2011) and Ferreira et al. (2018). Table 1 presents the centesimal composition of the dairy dessert samples. The moisture content was the parameter that offered the most significant variation between treatments, and the samples added with HC presented significantly lower moisture (*p* < 0.05) than the control sample. However, there was no significant difference between F3 (46.4%) and F4 (45.7%) (*p* > 0.05).

**TABLE 1:** Proximate composition.

Sample	Moisture	Fat	Protein	Ashes	Carbohydrates
F1	57.5 ± 0.62ª	6.43 ± 0.1^a^	7.8 ± 0.1^d^	3.93 ± 0.1ª	14.76 ± 0.7ª
F2	47.6 ± 1.4^b^	5.75 ± 0.2^ab^	9.0 ± 0.4^d^	3.88 ± 0.1^a^	14.75 ± 0.6^a^
F3	46.4 ± 0.37^cb^	5.00 ± 0.4^cb^	9.5 ± 0.5^dc^	3.93 ± 0.08^a^	13.58 ± 0.5^ba^
F4	45.7 ± 0.68^cb^	4.67 ± 0.3^c^	11.3 ± 0.3^cb^	4.03 ± 0.1^a^	12.68 ± 0.5^ba^
F5	44.7 ± 0.41^c^	3.90 ± 0.3^cd^	13.2 ± 1.2^b^	3.93 ± 0.05^a^	12.37 ± 0.07^b^
F6	41.1 ± 0.30^d^	3.00 ± 0.2^d^	15.6 ± 1.8ª	4.12 ± 0.4^a^	12.11 ± 0.4^b^

*Note*: Results are expressed as mean ± standard deviation. Different letters in the same column indicate a difference according to Tukey's test (*p* > 0.05). F1, F2, F3, F4, F5, and F6: dessert samples with 0%, 1%, 3%, 6%, and 9% HC, respectively. Results are expressed in %.

Fat content differed significantly between all treatments (*p* < 0.05) and followed the same behavior as moisture, decreasing with increasing HC addition, ranging from 6.43% (F1) to 3.00% (F6). Fat plays a fundamental role in the sensory characteristics, consumer acceptance, structural stability, and texture of several dairy products, and its low content can promote lower overall acceptance of the product by consumers (Zhao et al. 2023). However, compounds such as HC can act as fat substitutes in low‐fat food formulations as these compounds can improve the flavor and texture of the food (Sousa et al. 2017). The reduction in moisture and fat content observed in the HC‐containing samples (F2–F6) compared to the control (F1) can be attributed to the increase in dry matter resulting from the addition of HC, which raises the total solids content of the formulation (Essa and Elsebaie 2022). Moreover, some amino acids present in HC, such as glycine, proline, and hydroxyproline, can bind to water molecules through hydrogen bonding, promoting greater water retention during processing (Belitz et al. 2009). Previous studies have also demonstrated the hydration and water‐binding capacity of HC, reinforcing its role in influencing moisture content in food systems (León‐López et al. 2019; Kudo and Nakashima 2020). These characteristics reinforce the effects observed in this study, suggesting that HC not only contributes to a denser and more structured protein matrix but also acts as a structuring and water‐retention agent, capable of altering the distribution and availability of both moisture and fat in the product matrix.

Protein content of F1 and F2 did not differ significantly from each other (*p* > 0.05); however, after adding 3% HC (F3), the increase was gradual (on average 18% for every 3% CH), as expected, due to the high protein value of the HC (92.8%) (Gómez‐Guillén et al. 2011). Linear regression analysis (*R* ^2^ = 0.987) confirmed the direct relationship between HC concentration and protein content, with good fit and predictive capacity. The protein content results obtained in this research are essential for validating and classifying F5 and F6 samples as high‐protein products, according to the Technical Regulation on Supplementary Nutritional Information (National Health Surveillance Agency 2012), as they presented a protein content above 12 g/100 g, that is, 13.2% and 15.6%, respectively. There were no significant differences in ash content (*p* > 0.05); the ash and fat results are according to the results of León‐López et al. (2020) in their study of the addition of HC in fermented milk. However, they differed regarding protein content as they observed a percentage increase in the protein content of the sample after 1% addition of HC.

Regarding carbohydrate content, F1 and F2 showed statistically equal values (14.7%), whereas from sample F3 onwards, the treatments presented significant differences between them (*p* < 0.05). The similarity of the carbohydrate values of samples F1 and F2 is related to the fact that only 1% of HC cannot significantly alter this parameter as this compound is mostly composed of proteins (Gómez‐Guillén et al. 2011).

#### 3.1.2 Color

Color is one of the quality attributes that can directly affect product acceptance as appearance is one of the first factors that lead consumers to choose a product in a market (Pathare et al. 2013). Table 2 presents the color parameters of the dairy desserts, in which the addition of HC resulted in a significant reduction (*p* < 0.05) in values compared to the control sample (F1). F1 presented greater lightness (L* = 39.9), chroma (C* = 13.8), and hue (*h* = 49.1). The decrease in lightness in the HC treatments can be attributed to the darkening caused by the increase in free amino groups derived from collagen peptides during heating (Kumar et al. 2019), in addition to the lower fat content (Sousa et al. 2017; Cáceres et al. 2004).

**TABLE 2:** Color parameters.

Sample	L*	a*	b*		C*	*h*
F1	39.9 ± 0.1^a^	9.0 ± 0.07^a^		10.5 ± 0.2^a^	13.8 ± 0.2^a^	49.1 ± 0.2ª
F2	35.7 ± 0.04^b^	7.3 ± 0.1^b^		7.2 ± 0.2^b^	10.3 ± 0.3^b^	44.7 ± 0.4^cb^
F3	33.8 ± 1.2^c^	7.8 ± 0.1^b^		7.2 ± 0.1^b^	10.6 ± 0.1^b^	42.6 ± 0.9^dc^
F4	34.2 ± 0.2^cb^	7.4 ± 0.06^b^		7.3 ± 0.08^b^	10.4 ± 0.1^b^	44.7 ± 0.2^cb^
F5	32.5 ± 0.3^dc^	6.5 ± 0.05^c^		5.0 ± 0.03^c^	8.2 ± 0.05^c^	37.4 ± 0.5^d^
F6	31.0 ± 1.0^d^	5.5 ± 0.3^d^		4.5 ± 0.6^c^	7.1 ± 0.7^d^	41.0 ± 4.4^dc^

*Note*: Results are expressed as mean ± standard deviation. Different letters in the same column indicate difference according to Tukey's test (*p* > 0.05). F1, F2, F3, F4, F5, and F6: dessert samples with 0%, 1%, 3%, 6%, and 9% CH, respectively. *L*, a*, b*, C*, *h*, indicates brightness, red/green coordinate, yellow/blue coordinate, chroma, and hue angle, respectively.

The chroma value (C*) indicates color saturation, and the lower value observed in F6 (7.1; *p* < 0.05) indicates a less intense color, possibly masked by the cocoa, which has a dark hue. Among the samples with 1%–6% HC (F2–F4), no significant differences were observed in the red (a*), yellow (b*), and chroma (C*) parameters (*p* > 0.05), suggesting that these concentrations were not sufficient to visibly alter the color, given the strong influence of cocoa (Li et al. 2015; Cinar et al. 2021).

Understanding the masking effect of cocoa is crucial, especially when compared to other matrices, such as frankfurter‐type sausages, in which the influence of HC on color is generally more evident (Sousa et al. 2017). The impact of HC on color was observed to become more pronounced at higher concentrations, as in the case of sample F6, which presented significantly lower values for L*, a*, b*, C*, and *h* (*p* < 0.05). Similar results were reported by Sousa et al. (2017), who identified increased yellowing in products with higher HC content, reflecting the intrinsic coloring characteristics of this ingredient.

### 3.2 Microstructural Analysis

**FIGURE 1:**
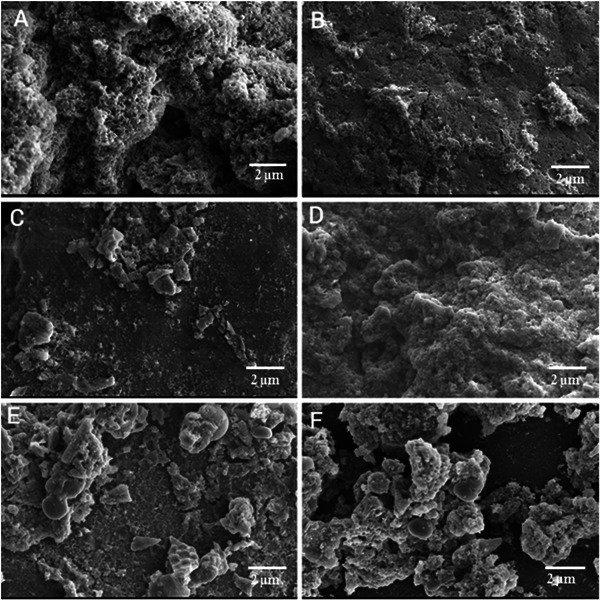
Scanning electron microscopy (SEM) at 5.00k× magnification and 10 kV resolution of dairy dessert samples with different concentrations of hydrolyzed collagen. A—F1 (control); B—F2 (1%); C—F3 (3%); D—F4 (6%), E—F5 (9%), and F—F6 (12%).

Figure 1 shows the microstructure of the different dairy dessert treatments. The gel formation in the dairy dessert is a result of the interaction between the proteins and xanthan gum under heating through the formation of complexes via electrostatic interaction, which can absorb large amounts of water while maintaining structural integrity (Le et al. 2017). HC can interact electrically with the gum and stimulate the formation of a double helix in the polysaccharide structure (da Trindade Alfaro et al. 2013). In addition, Laneuville et al. (2006) reported that whey proteins and xanthan gum are synergistic for gelation at low concentrations (0.1 g/100 g).

**FIGURE 2:**
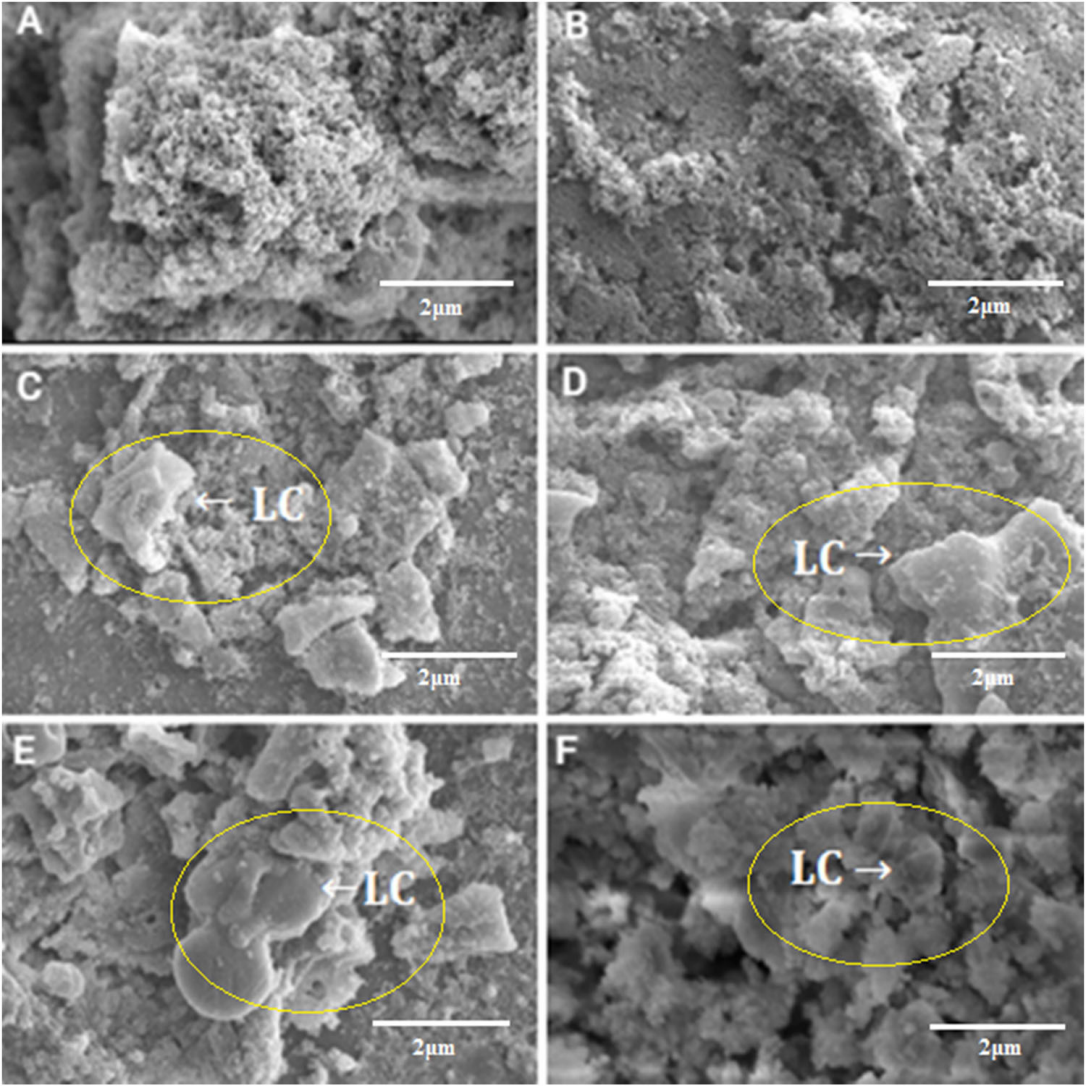
Scanning electron microscopy (SEM) at 10.00k× magnification and 10 kV resolution of dairy dessert samples with different concentrations of hydrolyzed collagen. A—F1 (control); B—F2 (1%); C—F3 (3%); D—F4 (6%), E—F5 (9%), and F—F6 (12%). LC, lactose crystals.

The micrographs (Figure 1) illustrate that the gel became more compact with increasing concentration of added HC, with F6 being the sample with the most evident change (Figure 1B–F). In Figure 2 (10.00k× and 10 kV resolution), it is possible to observe the formation of lactose crystals (C–F), which intensified with increasing HC concentration. A lactose supersaturation zone may result from adding HC and interacting with the solvent to form the gel (Pandalaneni and Amamcharla 2018). Darmali et al. (2021) described the influence of impurities on lactose crystallization, finding that the presence of whey protein significantly affected the lactose nucleation rate (*p* < 0.05) and reduced the nucleation time by about half compared to pure lactose. However, despite the noticeable microstructural difference between samples F1, F2, and F6, samples F3, F4, and F5 did not demonstrate any visual disparities (Figures 1 and 2).

### 3.3 Rheological Analysis

**FIGURE 3:**
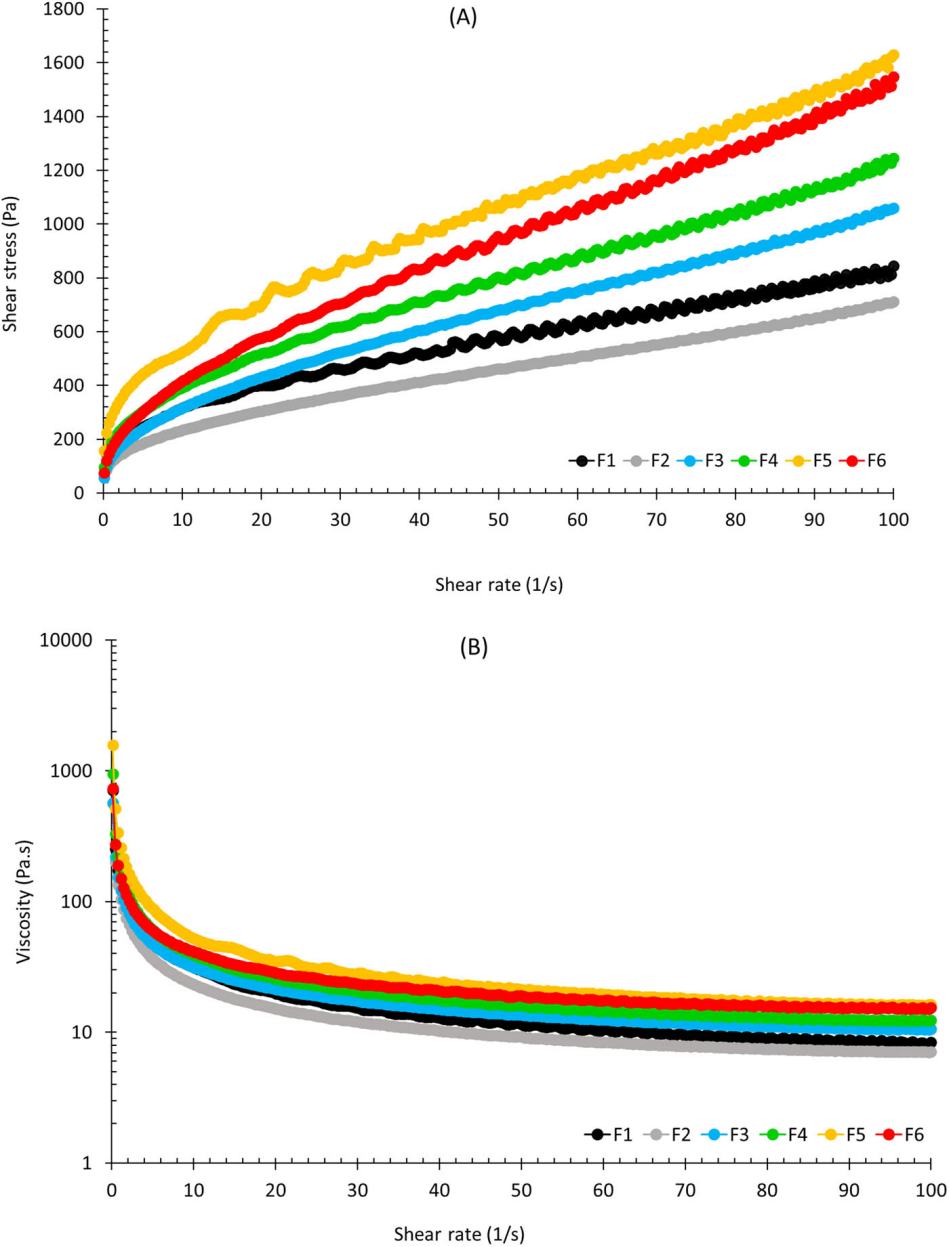
Steady‐state flow curves (A) and viscosity (B) of the samples F1–F6.

Figure 3A,B shows the flow curves and viscosity data obtained from the shear stress scans of the samples. In Figure 3A, the flow curves of all samples showed a similar profile equivalent to the behavior of pseudoplastic fluids, in which the increase in shear stress decreases with the increase in shear rate. This rheological profile is advantageous during industrial processing, as it facilitates operations such as pumping, filling, and mixing ingredients. From a sensory perspective, it is also desirable, as it contributes to a creamy, palatable texture (Alam et al. 2024). In Figure 3B, this behavior became clearer through the sharp decline in viscosity with increasing shear rate. The data were adjusted by nonlinear regression to the Herschel–Bulkley model (0.995 < *R* ^2^ < 0.998), and the estimated rheological parameters, yield stress (*σ*_0_), consistency index (*k*), and flow behavior index (*n*) are present in Table 3. The sample F5 exhibited yield stress (252 Pa) significantly higher than F4 and F6 (174–155 Pa), which were also significantly higher than the samples F1, F2, and F3 (119–110 Pa). In this regard, it seems the applied treatment had a huge impact on the protein network structure, increasing yield stress with an increase in the process parameters. Similarly, the F5 sample showed the highest consistency (59.3 Pa s*^n^*), but no significant differences were observed for the other samples (40.2–47.2 Pa s*^n^*), except for F2, which had the lowest consistency (22.9 Pa s*^n^*). This behavior suggests that the applied treatment may have caused an increase in the intermolecular forces of attraction between protein molecules and, consequently, promoted less mobility and higher consistency. All samples have shown a flow behavior index lower than 1 (0.582 ≤ *n* ≤ 0.738), indicating a shear‐thinning behavior typical of pseudoplastic fluids where there is a decrease in viscosity with shear rate, as can be seen in Figure 3B. Among all samples, F1 highlights the most pseudoplastic behavior (0.582), whereas F6 is presented as the least one (0.738). No huge differences were observed among the other samples.

**TABLE 3:** Herschel–Bulkley model parameters obtained from nonlinear regression of the flow curves of the F1–F6 samples.

Sample	*s*_0_ (Pa)	*k* (Pa s*^n^*)	*n* (−)	*R* ^2^
F1	119 ± 5.00^c^	47.2 ± 2.105^b^	0.582 ± 0.009^c^	0.995
F2	110 ± 2.86^c^	22.9 ± 0.880^c^	0.700 ± 0.008^ab^	0.997
F3	114 ± 3.79^c^	40.2 ± 1.240^b^	0.678 ± 0.006^b^	0.998
F4	174 ± 4.85^b^	40.3 ± 1.483^b^	0.702 ± 0.007^ab^	0.997
F5	252 ± 6.79^a^	59.3 ± 2.257^a^	0.672 ± 0.007^b^	0.997
F6	155 ± 5.57^b^	44.5 ± 1.540^b^	0.738 ± 0.007^a^	0.998

*Note*: Results are expressed as mean ± standard deviation. Same letters in the same column means significant no difference (*p* < 0.05) between samples according with the Student *T*‐test. *k* = consistency index; *n* = flow behavior index.

**FIGURE 4:**
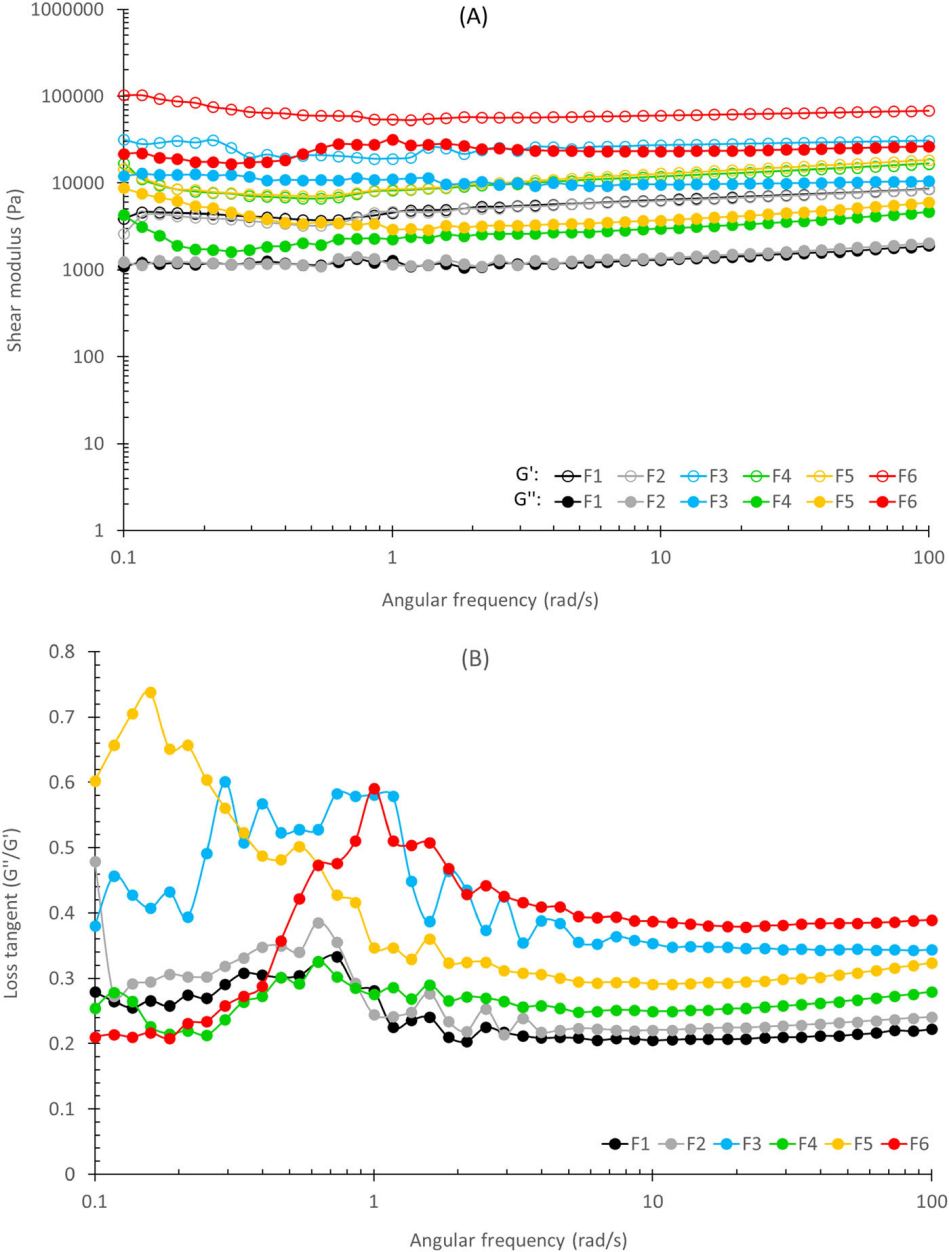
Shear modulus (A) and loss tangent (B) of the samples.

Figure 4A exhibits the mechanical spectrum of the samples. The shear modulus (*G*) of a material is the quantification of the resistance of the material against deformation in which *G*′ is related to the elastic response and *G*″ is related to the viscous response of the viscoelastic material (Ferrão et al. 2018). It is possible to notice that increasing frequency did not cause a significant disturbance in *G*′ and *G*″ profiles. The graphs have shown *G*′ ranging from 2500 to 103,000 Pa and *G*″ ranging from 1000 to 32,000 Pa. F1 and F2 have shown the lowest values of *G*′ and *G*″, whereas F5 and F6 had the highest. Overall, all samples have shown higher storage modulus than loss modulus, indicating that all of them had a more solid‐like gel behavior. The other samples had higher *G*′ and *G*″ in comparison to the F1 and F2 samples, suggesting that the treatment had a remarkable impact on the molecular structure of the protein network. Figure 4B shows the loss tangent, a ratio between the viscous (*G*″) and elastic (*G*′) properties, relating the relaxation of the bonds in the food matrix that are formed by milk proteins (Frohlich‐Wyder et al. 2009). All samples showed tan < 1, indicating *G*′ values higher than *G*″ across the whole frequency range, suggesting the samples can be interpreted as solid‐like gels with a well‐structured protein network (Cunha et al. 2012). Loss tangent values varied a lot but were more stable at frequencies higher than 10 rad/s, tending to constant values as frequency increases. Among the samples, there was a tendency to increase in loss tangent with the intensification of the treatment; however, the same pattern of increase was observed for the storage and loss moduli mentioned previously. Generally, this indicates that with enough time for relaxation between the bonds at the food matrix, the samples tend to have a typical solid‐like behavior. This rigidity and reinforced organization of the protein matrix are supported by microstructural analyses obtained by SEM, where it was possible to observe that the gel becomes progressively more compact as the HC concentration increases, with sample F6 presenting the most evident structural changes.

### 3.4 Sensory Analysis

#### 3.4.1 Acceptance Test

Table 4 presents the overall acceptance of dairy dessert samples. There were no significant differences in the overall acceptance of F1–F4 samples (*p* > 0.05), suggesting concentrations above 6% HC do not influence this parameter. The samples least accepted sensorially were F5 (5.529) and F6 (6.083), and they did not present significant differences. In a study of adding HC in fermented milk beverages, Znamirowska et al. (2020) reported that up to 3% addition of HC was also not enough to influence the overall acceptance of the product as, in low concentrations, HC does not influence essential aspects such as flavor and aroma.

**TABLE 4:** Frequency of use of check‐all‐that‐apply (CATA) terms and citation frequencies (%) for significant attributes (*p* ≤ 0.05), according to Cochran's *Q*‐test and general acceptance of the samples.

Attribute	Control	HC 1%	HC 3%	HC 6%	HC 9%	HC 12%	Ideal
Brown	84^d^	61^c^	40^b^	22^b^	30^b^	1^a^	20
Dark brown	22^a^	48^b^	67^bc^	88^c^	76^c^	113^d^	75
Presence of particles	41^ab^	45^b^	41^ab^	60^b^	44^b^	23^a^	1
Consistent appearance	21^a^	54^b^	64^bc^	61^bc^	53^b^	75^c^	65
Coffee aroma	3^a^	14^ab^	11^ab^	16^b^	8^ab^	10^ab^	15
Sweet aroma	53^c^	39^abc^	46^bc^	34^ab^	33^ab^	24^a^	51
Chocolate taste	83^b^	87^b^	89^b^	73^ab^	63^a^	75^ab^	110
Sweet	58^b^	60^b^	51^ab^	42^ab^	35^a^	35^a^	88
Bitter	34^a^	48^ab^	51^ab^	66^bc^	60^bc^	72^c^	41
Milk taste	39^c^	28^abc^	31^bc^	25^abc^	14^a^	16^ab^	34
Consistent	23^a^	55^bc^	82^d^	75^cd^	52^b^	77^d^	72
Creamy	94^c^	81^bc^	75^bc^	67^b^	67^b^	45^a^	101
Elastic	13^a^	19^ba^	19^a^	14^a^	28^a^	61^b^	14
Presence of lumps	53^ab^	72^bc^	63^ab^	92^c^	68^ab^	50^a^	2
Homogeneous	50^b^	37^a^	42^ab^	25^a^	35^ab^	52^b^	97
Overall linking	6702^ab^	6810^a^	6752^a^	6380^ab^	5529^c^	6083^bc^	—

*Note*: Results are expressed as citation frequency (%). Different letters in the same row indicate significant differences (p ≤ 0.05) between samples according to Cochran’s Q test followed by a multiple comparison procedure. Abbreviation: HC, hydrolyzed collagen.

#### 3.4.2 Check‐All‐That‐Apply

According to Cochran's *Q*‐test, the CATA terms selection frequency by the panelists was significantly different among the six formulations evaluated in this study (*p *< 0.05), suggesting that consumers perceived differences in the sensory characteristics of the desserts, except “shininess” (*p* = 0.267), “chocolate aroma” (*p* = 0.163), “sandy appearance” (*p* = 0.167), and “viscosity” (*p* = 0.198). In the description of the dessert samples, the terms “chocolate flavor,” “creamy,” “dark brown color,” “presence of lumps,” and “consistent” were the most cited, all above 45% (Table 4).

**FIGURE 5:**
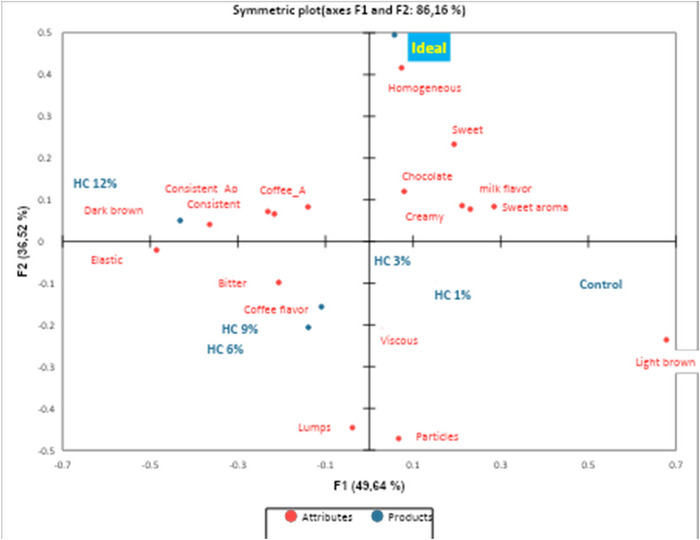
Correspondence analysis (CA) of samples, ideal product, and descriptors.

In the CA of the sensory profiles (Figure 5), dimension 1 represented 49.64% of the data, whereas dimension 2 represented 36.52%, explaining a total of 86.16% of the data variability. The sample with 12% HC was located in Dim 1 (−), and characterized by the attributes “consistent appearance,” “coffee aroma,” “dark brown color,” and “consistent.” The samples with 6% and 9% HC and PB were located in Dim 2 (−) and were associated with the attributes “elastic,” “coffee flavor,” “bitter,” and “presence of lumps.” Control samples, with 1% and 3% HC, were located in Dim 2 (+), presenting attributes such as “light brown color,” “viscous,” “presence of particles,” “homogeneous,” “sweet,” and “chocolate flavor.” In general, the six dessert samples presented a good distribution of attributes among the four quadrants, with the sample added with 3% HC being the closest to an ideal dairy dessert.

Penalty analysis determines how much the overall acceptance is reduced by deviations in sensory profiles between real and ideal products (Wang et al. 2023), in addition to allowing the selection of essential attributes or those that negatively affect the sensory evaluation of the product (Galli et al. 2019). In the present study, the attributes “chocolate flavor,” “sweet,” and “creamy” were classified as “essential/mandatory” for the dessert. The control sample presented the highest frequencies for these attributes, and samples F2 and F3 presented the highest frequencies compared to F4, F5, and F6 samples, suggesting that HC concentrations above 3% are indicated to maintain the preferred sensory profile. Rigoto et al. (2019) obtained similar results in probiotic dairy drinks produced with HC, demonstrating that the addition of 1% HC did not affect the aroma, flavor, and texture of this product. “Consistent appearance” was also indicated as “essential/mandatory,” and sample F6 showed its highest frequency, due to the higher concentration of HC and the increased gelling property resulting from the interaction of the protein with hydrocolloids, fat, and water in the product (Le et al. 2017). The attributes “dark brown color,” “consistent,” and “homogeneous” were classified as “do not influence preference.” In contrast, the other terms, except “bitter,” were classified as “do not harm the product.” The attribute “bitter” was classified as negative/undesirable. F6 sample also showed the highest frequency for this parameter, possibly due to its lower carbohydrate and fat contents (Rigoto et al. 2019). Furthermore, in certain pH variations (in the case of the present study, possibly promoted by the added protein), cocoa can promote acidity, astringency, and bitterness, resulting from polymerization reactions, oxidation of polyphenols, and increased activity of the polyphenol oxidase enzyme (Valverde Garcia et al. 2020).

Morais et al. (2015) evaluated probiotic chocolate dairy desserts. They observed that the chocolate flavor, sweet taste, and aroma attributes were determinants in product preference, whereas bitterness and bitter aftertaste were the negative drivers. In agreement, Cutrim et al. (2023) concluded that flavor is the most relevant attribute in the purchase decision of a dairy dessert, as for each increase of 1 U in the acceptance of the flavor, the probability of purchase increases by 3.44 times. Thus, it is possible to conclude that although HC presents important texture properties and great potential to replace fat in dairy desserts, additional studies are still needed to understand the effects of adding HC in concentrations above 9% on the flavor of the product.

Among the samples evaluated, F3 presented the closest approximation to the ideal sensory profile, being associated with desirable attributes such as “chocolate flavor,” “sweet,” and “creamy.”

## 4 Study Limitations

Although the results obtained are promising, some methodological limitations should be highlighted. Microstructural characterization was based exclusively on visual analyses using SEM, without the use of complementary quantitative techniques, which may restrict the accurate interpretation of the structural changes induced by HC. Regarding sensory aspects, the presence of lumps and particles in some samples may be related to the difficulty in homogenizing formulations with higher total solids content (Lopes 2023). Furthermore, the lack of specific sensory training and reference products may have influenced the participants’ interpretation and assignment of sensory terms, as previous experiences with commercial dairy desserts, generally higher in sugar and fat, possibly influenced their perceptions (Bhuker and Maurya 2024). The evaluator's age group was also a critical factor in the study, as younger individuals generally prefer sweeter flavors (Dos Santos et al. 2017). This reflects the need for future studies that combine more comprehensive microstructural analyses and the use of trained sensory panels in order to obtain a more robust characterization of low‐calorie functional dairy desserts.

## 5 Conclusion

HC increased protein content, with samples F5 and F6 considered high‐protein products according to legislation, in addition to reducing moisture and fat parameters due to the increased dry matter content. This effect is relevant for the formulation of desserts with a protein‐rich and reduced‐calorie content. HC also influenced the microstructure and rheology parameters of the samples, with higher concentrations resulting in more compact gels, and samples F3, F4, F5, and F6 demonstrating higher *G*′ and *G*″ compared to samples F1 and F2, suggesting that HC concentrations above 9% exert a more significant impact on the molecular structure of the protein network. This behavior can be exploited by the industry to adjust the texture, consistency, and stability of dairy desserts and other similar functional products. Overall acceptance showed a significant reduction only after the addition of 9% HC, whereas formulation F3 was associated with the sensory attributes “chocolate flavor,” “sweetness,” and “creaminess,” configuring it as the closest to the ideal dairy dessert profile. In summary, the results indicate that the addition of 1%–3% HC favors the balance between structural characteristics and sensory acceptance in dairy desserts, highlighting the potential of HC as a functional ingredient. Despite the need for future studies on a pilot or industrial scale with trained sensory panels, these results provide important insights for the development of innovative functional dairy products.

## Author Contributions

**Maria Eduarda Marques Soutelino**: conceptualization, investigation, writing – original draft, methodology, formal analysis. **Bianca Cristina Rocha de Oliveira**: investigation, methodology. **Louise de Aguiar Sobral**: investigation, methodology, formal analysis. **Fábio Jorge de Vasconcellos Junior**: investigation, methodology, formal analysis. **Eliane Teixeira Mársico**: writing – review and editing, validation, visualization, project administration, supervision, resources. **Erick Almeida Esmerino**: methodology, validation, visualization, writing – review and editing, software, project administration, supervision. **Adriano Gomes da Cruz**: investigation, validation, methodology, project administration, supervision. **Adriana Cristina de Oliveira Silva**: conceptualization, writing – review and editing, visualization, validation, methodology, project administration, supervision.

## Conflicts of Interest

The authors declare no conflicts of interest.

## Data Availability

Data sharing is not applicable to this article.
